# Mind–Body Medicine Training for Incarcerated Men and Women

**DOI:** 10.3390/healthcare14060746

**Published:** 2026-03-16

**Authors:** Julie K. Staples, Jesse Rice, Kathleen S. Farah, Sabrina N’Diaye, James S. Gordon

**Affiliations:** 1 The Center for Mind-Body Medicine, Washington, DC 20015, USA; jstaples@cmbm.org (J.K.S.); kfarah@cmbm.org (K.S.F.); sabrina@theheartnest.com (S.N.); 2 Department of Biochemistry and Molecular & Cellular Biology, Georgetown University, Washington, DC 20057, USA; 3 College of Osteopathic Medicine, Kansas City University, Kansas City, MO 64106, USA; jesse.rice@kansascity.edu

**Keywords:** mind–body medicine, prisoners, incarcerated, meditation, resilience, mindfulness, education

## Abstract

**Background/Objective:** Mind–body programs teaching mindfulness-based techniques have benefits for incarcerated people, as do programs in which individuals teach yoga to their incarcerated peers. However, there are no studies of comprehensive programs that combine a variety of self-care techniques with group support and enable people in prison to enhance their own well-being and then share what they have learned with their peers. This study evaluated the effects of such a training program in the United States. **Methods:** Thirty-eight incarcerated men and women began the 8-day mind–body medicine training and 31 completed the training. Mind–body techniques taught included soft belly breathing, meditation, autogenics and biofeedback, guided imagery, mindful eating, self-expression through drawings and writing, and genograms. Outcomes included resilience, depression, anxiety, stress, coping self-efficacy, optimism, meaning in life, and purpose in life. Outcomes were measured before and after the training, and at a 6-month follow-up. **Results:** There were significant improvements in resilience, depression, anxiety, stress, coping self-efficacy, optimism, the presence of meaning in life, and purpose in life after the training. All of these improvements were maintained at follow-up. The most frequently practiced skills both after the training and at follow-up were soft belly breathing, meditation, and mindful eating. **Conclusions:** The training provided participants with skills that had a lasting positive benefit on numerous aspects of their own well-being and trained them to teach the skills to their incarcerated peers. The results of this uncontrolled study suggest that the mind–body medicine training program may be helpful to incarcerated people in other prison systems.

## 1. Introduction

Incarcerated people have higher levels of mental health disorders than the general population. James and Gaze in a United States (U.S) Bureau of Justice Statistics Report [1] found that 24% of people in a state prison had a recent history of a mental health problem while only 11% of the U.S. population met the criteria for mental health disorders. People living in prison also endure chronic stress. Stressors may include a lack of privacy, overcrowded conditions, antagonistic relationships with guards and incarcerated peers, the threat of personal violence, and witnessing violence [2]. In addition, incarceration disrupts healthy bonds with families and support systems [3]. Two meta-analyses of mindfulness-based interventions offered in prisons found a reduction in stress, depression, anxiety, and overall psychological distress [4], and an improvement in psychological well-being and behavioral functioning in incarcerated individuals [5]. Not only can mind–body programs help improve life while in prison, but two studies of yoga interventions show significantly reduced reincarceration rates 2 years [6], and up to 5 years, later [7].

The effectiveness of these mind–body approaches has resulted in their incorporation into prisons around the world [8]. Programs teaching these techniques to incarcerated populations are relatively common. However, there are only two published studies on 200 h yoga teacher training programs in prisons that train incarcerated men to teach yoga to others in prison [9,10]. These programs offer incarcerated people skills to enhance mental and physical well-being while in prison, and to potentially teach to other incarcerated people. They also equip those in prison with skills they might be able to use as part of post-incarceration employment and personal adjustment. Upon leaving prison, individuals can experience post-incarceration syndrome which includes post-traumatic stress disorder (PTSD) symptoms as well as difficulties in social interactions and engaging in relationships [11]. Meditation can reduce PTSD symptoms [12] found in post-incarceration syndrome. Yoga and mindfulness programs may also help post-incarceration social/relationship issues because relationship satisfaction is mediated by mindfulness and emotional intelligence which are acquired through these programs [13].

While the yoga and mindfulness-based programs that have been used in prisons are very helpful during and possibly after incarceration, they are not comprehensive. They do not provide a wide variety of mind–body techniques and tools for self-expression that act in different ways to enhance wellness, nor do they allow each participant to choose the ones that are most effective for him or her. They also do not provide the small-group support that facilitates the use of all the techniques and offers incarcerated individuals an opportunity to share the challenges they face as they deal with past trauma and present stress. The Center for Mind–Body Medicine (CMBM) has developed a comprehensive mind–body medicine training program that teaches participants how to deal with their own stress and trauma. The program then teaches them how to share what they have learned and benefitted from with others in small groups, as well as individually.

The training consists of lectures on, and the practice of, mind–body techniques including soft belly breathing, movement, mindfulness, and biofeedback, all of which help to restore the physiological and psychological balance, which has been disrupted by trauma and stress. Once that balance has begun to be restored, participants learn to use guided imagery, drawings, and genograms to help them mobilize their imagination, to enhance decision making, and to provide a greater understanding of themselves and others. The small groups, where confidentiality is required, are “safe places” in which the techniques are practiced and experience of them can be shared. They provide the social support which has been significantly and positively correlated with quality of life in incarcerated individuals [14].

The CMBM training program has been widely used to train both health care professionals in the U.S. [15] and Gaza [16] and teachers in Kosovo [17]. Adult participants of the mind–body skills groups facilitated by those trained in the program have experienced significantly reduced PTSD [16,18], depression, and anxiety, and an improved quality of life [16]. Resilience was significantly increased in nursing and compassionate care leaders in a hospital health system after taking this training [19]. Resilience is a trait that could help individuals endure the chronic stress of being in prison and has been shown to have a negative correlation with depression and anxiety in incarcerated women [20]. The mind–body medicine training might also be expected to improve stress, optimism, coping self-efficacy, and meaning and purpose in life based on the results of other mind–body programs. Yoga decreased stress [9] and Vipassana meditation increased optimism in incarcerated individuals [21]. Coping self-efficacy was increased in a program using mind–body techniques such as imagery and breath awareness [22]. The presence of meaning in life was higher in meditators than non-meditators [23] and purpose in life was increased following a 3-month meditation retreat [24]. While these benefits have been measured with other programs, this training has never been taught to an incarcerated population. The purpose of this uncontrolled study was to determine whether the mind–body medicine training program for incarcerated men and women would decrease depression, anxiety, and stress, and increase resilience, optimism, coping self-efficacy, and meaning and purpose in life.

## 2. Materials and Methods

### 2.1. Participants and Study Design

The participants in the Mind–Body Medicine Training Program (MBMTP) were recruited from two Indiana Department of Correction (IDOC) facilities in Indianapolis, Indiana, USA. Female participants were from the maximum-security Indiana Women’s Prison. Male participants were from the medium-security Plainfield Correctional Facility. The study design was a pre/post/follow-up study design without a control group. It was not possible to do a randomized controlled study due to funding limitations. IDOC staff time was required for recruiting and screening participants and for coordinating the training. Their time limitations did not allow for additional recruiting and data collection from a control group.

### 2.2. Recruitment

In order to introduce mind–body medicine and the training to the participants, 2 h workshops were held at each facility prior to the training. Workshops included presentations on the introduction to Mind–Body Medicine/Mind–Body Skills groups and a soft belly breathing experiential with sharing. There was also a presentation on movement and expressive meditation followed by the practice of these techniques and a question-and-answer session. Flyers with information about the research study were provided at the workshops and were accessible to the incarcerated individuals on their electronic tablets. Participants in the workshops were invited to contact their Unit Team Staff if they were interested in participating in the MBMTP research study. The Unit Team Staff collected the names of those interested and provided the names to the Executive Director of Behavioral Health, who held an informal interview with interested participants to determine their eligibility.

Inclusion criteria were English-speaking incarcerated individuals at both of the above facilities. There were two exclusion criteria. The first was having any of the following: completion of their prison sentence; a scheduled change in security level to a different prison; or a scheduled work release before the study was going to end. Also excluded were those having conduct problems and/or emotional or mental instability that prevented safe and effective participation in the MBMTP.

### 2.3. Mind–Body Medicine Training Program

The MBMTP has two parts, each 4 days long. In the women’s prison, the two parts of the training were held 1 month apart in November and December 2022. In the men’s prison, the trainings were held 3 months apart in May and August 2023 due to COVID-19 outbreaks in the prison. Details on the lecture topics and the skills practiced are outlined in Table 1.

The 4-day Initial Training consisted of both lectures and participation in two small groups per day. In the small groups—each of about 10 people—the participants practiced the mind–body skills they were taught and shared their experience of the practice. Three small-group sessions were dedicated to genograms. Genograms are drawn like family trees, and also include information about relationships and interactions among family members, as well as family patterns of employment and illness, and challenges that recurred over generations. This exercise encouraged participants to explore and make imaginative use of strengths, vulnerabilities, and sources of emotional support, in their own families of origin as well as in families of choice.

The second 4-day Advanced Training gave trainees the opportunity to facilitate small groups in which they taught the skills they had learned to incarcerated peers who were also participating in the program. They did this under supervision of CMBM faculty who provided feedback sessions during and following each group session. The large-group meetings that were part of this training provided trainees with the opportunity to discuss such challenging issues as withdrawn or angry participants, and offered a demonstration by CMBM faculty of leading a small group.

### 2.4. Measures

Assessments were conducted at baseline (T0), at the end of the Advanced Training (T1), and at 6-month follow-up (T2). Resilience was the primary outcome measure and was measured using the Connor–Davidson Resilience Scale (CD-RISC). The CD-RISC consists of 25 questions that are rated on a 5-point scale from 0—“not true at all” to 4—“true nearly all of the time”. The total score range is 0–100. Higher scores represent greater resilience. The CD-RISC has shown good internal consistency (Cronbach’s α = 0.89) [25]. In this study, α = 0.82 for the baseline values.

Depression, anxiety, and stress symptoms were measured using the Depression Anxiety and Stress Scale-21 (DASS-21). The DASS-21 consists of seven questions each to measure the emotional states of depression, anxiety, and stress. The questions are rated on a 4-point scale from 0—“did not apply to me at all” to 4—“applied to me, very much or most of the time”. Scores range from 0–21 for each of the subscales. Higher scores indicate higher levels of these symptoms. The DASS-21 has been shown to have good reliability with internal consistencies as follows: Depression (α = 0.88), Anxiety (α = 0.82), and Stress (α = 0.90) [26]. In this study, the baseline values had an α = 0.89 for depression, α = 0.72 for anxiety, and α = 0.81 for stress.

Optimism was measured with the Revised Life Orientation Test (LOT-R), which is a revised version of the original form of the Life Orientation Test. The LOT-R consists of 10-items that are rated using a 5-point scale from 0—“strongly disagree” to 4—“strongly agree”. Four of the items are filler items and are not included in the total score. The total score ranges from 0–24. Higher scores signify greater optimism. The LOT-R has been shown to be reliable with acceptable internal consistency α = 0.78) [27]. The baseline internal consistency for this study was α = 0.86.

The Coping Self-Efficacy Scale (CSES) was used to measure changes in the study participants’ perceived confidence in their ability to cope effectively with life challenges. The CSES consists of 26 items that are rated on an 11-point scale from 0—“cannot do at all “ to 10 “certain can do”. The total score ranges of 0–260 with higher scores reflect greater coping self-efficacy. The CSES has very good reliability (α = 0.95) [28]. In this study, the baseline internal consistency was α = 0.96.

The CSES has three subscales [28]: problem-focused coping; emotion-focused coping; and social support coping. Problem-focused coping measures a person’s self-efficacy to use cognitive strategies to make problems less severe. This subscale contains 6 items with a score range of 0–60. Emotion-focused coping evaluates one’s self-efficacy in altering emotional responses to unsettling events or problems, rather than addressing the problem itself. This subscale has four items with a score range of 0–40. Social support coping assesses an individual’s perceived ability to seek help from friends and family to cope with problems. It has three items and a score range of 0–30.

The Meaning in Life Questionnaire (MLQ) was used to measure presence of meaning in life (MLQ-P) and search for meaning in life (MLQ-S). The MLQ is a 10-item questionnaire with a 7-point scale from 1—“absolutely untrue “to 7—“absolutely true”. Each subscale has five items and a range of 5–35 points. Higher scores indicate a greater presence of, or a greater search for, meaning in life. The MLQ has shown good internal consistencies for both MLQ-P (α = 0.86) and MLQ-S (α = 0.92) in university and college students [29]. The baseline Cronbach’s α for the MLQ-P in this study was α = 0.91 and, for the MLQ-S, α = 0.88.

Purpose in life was measured using the Life Engagement Test (LET). The LET is a 5-item scale from 1—“strongly disagree” to 5—“strongly agree”. The total core range is 6–30 points with a higher score reflecting a greater sense of purpose in life. The LET was found to have an acceptable internal consistency α = 0.72 to 0.87 (average α =0.80) [30]. In this study, the baseline α = 0.80.

The frequency of mind–body skills practiced by the participants was measured using a questionnaire with 7 questions asking how often they had been practicing the skills. Each skill was listed, followed by time choices. The number of days per month used for the analysis are as follows: Never (0), Less than once a month (0.5), Once a month (1), Twice a month (2), Once per week (4.3), 2–3 times per week (10.85), 4–6 times per week (21.7), and Daily (30.4). This questionnaire was administered after the training and at 6-month follow-up.

### 2.5. Statistical Analysis

An a priori sample size calculation was not done because the number of participants to be trained was determined by the amount of funding for the program. A post hoc power analysis was done using data using our primary outcome measure, resilience, with G*Power version 3.1.9.7 [31]. Using a sample size of 30 (the matched T0 and T1 data) and α = 0.05, Cohen’s *d* was 1.237 with 0.99 power.

There was very little missing data (0.1%). In total, 10 of 9683 questions from the outcome measures were unanswered. Eight participants did not answer 1 question and one participant did not answer 2 questions. One participant refused to fill out many pages/questions of the research packets so only the completed outcome measures from this participant were included in the analysis. The 10 missing values described above were imputed using SPSS Version 29 (IBM, SPSS). Multiple imputations using a linear regression model were performed. The data was scanned and the automatic function determined the imputation method. For the outcomes with one missing datapoint, the monotone method was used. For the outcomes with more than one missing datapoint, the fully conditional specification (FCS) was used. Ten imputations were performed for all imputations on each outcome with missing values.

The MIXED procedure in SPSS was used to run a linear mixed model. A marginal model was used to account for the repeated measures over time for individuals. An unstructured covariance matrix of the residuals was used in this model. Time was a fixed factor. In addition, the following were included as fixed factors as well as their interactions with time: gender, age, race, and time served. Age and time served were categorized and the categories were included in the models. For age, the categories were in 10-year increments. For length of time served, the categories were as follows: 5 years or less, 6–19 years, and 20 or more years. If any of the fixed factors or their interactions were significant, they were included in the final model. Pairwise comparisons of differences between timepoints were performed using a Sidak correction.

The Wilcoxon signed-rank test was used to determine whether there was a median difference in the practice of each mind–body skill between the T1 and T2 timepoints. The exact sign test was used instead for breathing and meditation because the distribution of the differences between the timepoints was not symmetrical. Spearman’s correlation was used to determine the association between the frequency of the practice of the mind–body skills and the amount of change in the scores of the outcome variables.

Statistical analysis was performed to test for differences between participants who completed the study (*n* = 23) and non-completers (*n* = 23). Non-completers including the following: never began the training (*n* = 8), began but did not complete the training (*n* = 7), or completed the training but did not participate in the follow-up data collection (*n* = 8). Due to outliers or data that was not normally distributed, a Mann–Whitney U was run to determine if there were differences between baseline scores of DASS-21 depression, LOT-R, LET, and the length of the prison sentence, time served, and time remaining on the prison sentence in the completer and non-completers. For all other baseline outcome measure scores and age, independent-sample *t*-tests were done for testing differences between completers and non-completers. Fisher’s Exact test was used to test for differences in gender between completers and non-completers. A chi-square test of independence could not be used to test for differences in race due to expected counts less than five. Therefore, the “other” category was not included in the race data and the Fisher’s Exact test was run instead. A Mann–Whitney U test was run to determine if there were differences in the length of the prison sentences, time served, or time remaining on the prison sentences between men and women.

## 3. Results

### 3.1. Participant Flow and Characteristics

Forty-six subjects consented and completed the baseline questionnaires. Eight subjects did not begin the training: three had no reason given; two opted out of participating in another IDOC program; one asked to be removed; one was released before training began due to COVID delays; and one was transferred to another facility. Thirty-eight began the training but seven did not complete it for the following reasons: two were released; two for reasons not known; two had conduct problems; and one was moved to restrictive housing. Thirty-one completed the training. Eight were lost to follow-up because three were transferred to another facility; three were released; one did not attend the session to complete the follow-up questionnaires; and one did not fill out most of the questions. Data included in the analysis were as follows: the baseline data from all consented 46 participants, the post data from the 31 who completed the training, and follow-up from the 23 remaining. The characteristics of the 46 participants are shown in Table 2.

### 3.2. Outcome Measures

There were significant improvements in resilience, depression, anxiety, stress, optimism, coping self-efficacy (total score, problem-focused coping, emotion-focused coping, and social support coping), presence of meaning in life, and purpose in life after the training as shown in Table 3. There was no significant change from post to 6-month follow-up in these outcome measures, indicating that improvements were maintained at follow-up. Follow-up scores in all of these outcomes also remained significantly improved compared to baseline. There was no significant improvement in the search for meaning in life. Cohen’s d effect sizes from T0 to T1 were as follows: resilience, depression, and problem-focused coping d = 1.2; anxiety d = 0.77; stress d = 0.96; optimism d = 0.95, coping self-efficacy total score and emotion focused coping d = 1.3; social support coping d = 0.94; presence of meaning in life d = 0.94; and purpose in life d = 0.96.

Emotion-focused coping self-efficacy was the only outcome measure where the amount of time served had a significant main effect. Participants who had served 20 or more years on their sentence had significantly higher levels of emotion-focused coping self-efficacy across all timepoints (−1.7 [95% CI, −3.5, −0.033], *p* = 0.045) compared to those who had served 5 years or less. These results indicate that, after the training and at the 6-month follow-up, those who had been in prison longer had a greater level of belief that they could alter their emotional responses to stressful situations.

### 3.3. Practice of Mind–Body Skills

Twenty-three participants with matched post and follow-up data on their practice of the mind–body skills were included in the analysis. The frequency of practice is shown in Table S1. The most frequently practiced skills both after the training and at follow-up were soft belly breathing, meditation, and mindful eating. The median practice from T1 to T2 with statistical significance values is shown in Table 4.

There was no statistically significant median decrease in the frequency of practice of meditation, imagery, mindful eating, biofeedback/autogenics, or dialogue with a symptom/problem (an imagery/writing exercise) at T2 compared to the frequency of practice at T1. These results indicate that the participants continued to practice these mind–body skills 6 months after the program at about the same frequency as they did at the end of the training.

Although there was a statistically significant decrease in the median practice of breathing and movement at T2 compared to T1, breathing was the most frequently practiced skill with nearly 70% practicing it daily after the training. It was also still widely practiced 6 months after the training with 78% of participants doing the soft belly breathing skill at least 2–3 times a week (Table S1). There was an overall downward shift in the frequency of practice of movement, specifically shaking and dancing at T2. However, the participants had only sporadic access to music, so it may not have been possible to continue to easily practice this skill.

### 3.4. Correlation of the Practice of Mind–Body Skills with Outcome Improvements

Table 5 shows the results of the correlation analysis where there was a significant association between the frequency of practice of the mind–body skills and improvement in the outcomes. After the training, the increased frequency of practice of soft belly breathing was associated with an improvement in resilience (*r_s_* = 0.410). At follow-up, soft belly breathing practice was associated with an improvement in depression (*r_s_* = −0.429) and stress (*r_s_* = −0.435). Meditation (*r_s_* = 0.503) and soft belly breathing practice (*r_s_* = 0.491) were both significantly associated with an increased sense of purpose in life at follow-up.

### 3.5. Completer Versus Non-Completer Analysis

Completers had statistically significantly higher levels of baseline stress and search for meaning in life compared to non-completers (Table S2). None of the other baseline outcome scores were significantly different. There was no significant difference in the race of those who completed the study (46.4% of White participants completed; 63.6% of Black participants completed) (*p* = 0.271). There was also no significant difference in average age (completers 39.1 years; non-completers 37.2 years) (*p* = 0.475). However, completers had significantly longer prison sentences and significantly more time remaining on their sentences, and had served significantly more time than non-completers. It is possible these participants were more dedicated to completing the training and participating in the data collection because they were going to be in prison longer and had more expectation that the program would help them reduce their stress and find meaning in life. In addition, significantly more women completed the study (*p* = 0.003). Seventy-five percent of the women in the study were completers whereas only 30% of the men in the study were completers.

Both gender and the variables related to the prison sentences were significantly different between completers and non-completers. Therefore, an additional analysis was done to determine whether there was a significant difference in the prison sentences, time served, and time remaining in the sentence between men and women. Women had significantly longer prison sentences (median (*Mdn*) = 18 years) than men (*Mdn* = 9.5 years), *U* = 146, *z* = −2.539, *p* = 0.011. As would be expected because of the longer sentences, women also had statistically significantly more time left on their sentences (*Mdn* = 14.5 years) than men (*Mdn* = 5.5 years), *U* = 96.5, *z* = −3.459, *p* = <0.001. However, the time served on the sentences was not significantly different between women (*Mdn* = 4.9 years) and men (*Mdn* = 4.3 years), *U* = 209, *z* = −1.131, *p* = 0.258. It is not possible to tell whether more women completed the study because the training may have appealed more to women in general; whether the longer prison sentence was the reason they were more likely to complete the study; or whether the drop-out rate was affected by something that was not measured.

## 4. Discussion

Participation in the MBMTP resulted in significant improvements in resilience, depression, anxiety, stress, coping self-efficacy, optimism, the presence of meaning in life, and the sense of purpose in life in both incarcerated men and women. The improvements in all of these outcomes were maintained at the 6-month follow-up. Soft belly breathing, meditation, and mindful eating were the most frequently practiced skills after the training and at follow-up. Participants maintained their practice after the training and continued to practice meditation, imagery, mindful eating, and dialogue with a symptom/problem at about the same frequency at follow-up. There were significant positive correlations with soft belly breathing and improvements in resilience, depression, stress, and purpose in life. Meditation also had a significant correlation with an increased sense of purpose in life.

The MBMTP improved resilience which is a predictor of high purpose in life scores [32]. High purpose in life scores are significantly lower in incarcerated people than in non-incarcerated people [33]. This was found in the present study. The incarcerated purpose in life scores before the training (*M* = 22.3, *SE* = 0.7) were lower than normative scores of a community sample (*M* = 25.1, *SE* = 0.3) [27]. The incarcerated scores were significantly improved after the training, and then were nearly the same (*M* = 25.8, *SE* = 0.7) as the normative scores [27]. The enhanced sense of purpose was reflected in statements by several of the trainees who said what they had learned had given them a sense of purpose and that they planned to use the skills in their communities when they left prison.

The improvement in the presence of meaning in life following the training is also important because the distress of people living in prison is predicted by a loss of meaning in life [34]. After the training, the presence of meaning in life scores were greater than student norm scores (*M* = 29.5, *SE* = 1.1 vs. *M* = 24.0, *SE* = 0.5) [29]. Incarcerated people with a high presence of meaning in life have less distress, more self-worth, enhanced care for others, and a more positive view of the world than those with a low presence of meaning in life scores [34]. These qualities were reflected in the participants’ enthusiasm for sharing what had benefitted them with other incarcerated people. Incarcerated individuals in this study were searching for meaning in life before the training as indicated by higher scores (*M* = 27.1, *SE* = 0.9) than the norm (*M* = 22.5, *SE* = 0.5) [29]. It is likely there was no significant increase in the *search* for meaning in life after the training because the training had in fact increased the actual presence of meaning in their lives.

The results from the coping self-efficacy scale showed that incarcerated individuals who have served more time on their sentences may have different coping styles and beliefs in their ability to cope. Emotion-focused coping self-efficacy “measures a respondent’s self-efficacy with respect to altering his emotional response to an unsettling event” [28]. Problem-focused coping is another way of coping that focuses on managing the problem that is causing the distress [35]. Problem-focused coping is a strategy that was often used by people serving less than two years in maximum-security prisons [36]. While problem-focused coping self-efficacy significantly improved in our study, we saw no difference between those serving short vs. long terms. This suggests that all participants built on their success in practicing and benefitting from the skills to deal more effectively with other challenges. However, emotion-focused coping self-efficacy was higher across all timepoints in those serving 20 or more years compared to those who served 5 years or less.

In a prison environment where there is limited control, those who have served the most time may have realized that their actions will not influence certain problems but emotion-focused coping strategies can reduce the stressfulness of these problems [35,36]. This may explain the significantly higher emotion-focused coping self-efficacy scores across all timepoints in those who served the longest time. Regardless of the time served, the emotion-focused coping self-efficacy scores were increased after the training and were maintained at follow-up. It is likely that the mind–body skills taught and the resulting decreases in anxiety and the attitude of mindful acceptance fostered, as well as the social support provided by the small groups, enhanced the emotion-focused coping self-efficacy.

One study on a training program using other mind–body interventions achieved similar results as the MBMTP. A study of a yoga teacher training program for incarcerated men found that participants had significantly decreased stress levels that were maintained at the 3-month follow-up [9]. A meta-analysis on a wide variety of mindfulness-based interventions for incarcerated populations reported on improvements in anxiety, stress, and depression [4]. The meta-analysis of five studies showed a moderate effect for improving depression for the following interventions: Acceptance and Commitment Therapy (ACT); Mindfulness-Based Stress Reduction (MBSR); Mindfulness-Based Cognitive Therapy (MBCT); Dialectical Behavior Therapy (DBT); and Transcendental Meditation (TM). Eight studies using these same interventions showed a small effect for anxiety reduction. There was also a large effect in reducing stress in three studies using MBSR, MBCT, or TM. Resilience was significantly increased in a study on a mantra-based stress reduction program for incarcerated Veterans [37] and optimism was increased in incarcerated men and women taking a Vipassana meditation course [21].

Some of these programs have several components such as yoga which includes posture, breath regulation, meditation, and ethics. However, they do not teach a variety of different types of techniques. One of the virtues of the MBMTP is that it is a comprehensive program teaching sixteen mind–body techniques and forms of self-expression. Participants are encouraged to discover which of the techniques are most appropriate for each of them, affording them an opportunity for choice and control, a welcomed change from the regimentation and constraints of prison life. An additional important difference in the studies which reported similar outcomes was that, with the exception of the yoga teacher training program, these programs were not taught to incarcerated individuals so they could, in turn, teach their peers.

The continued and frequent practice of a number of these techniques, including soft belly breathing, meditation, and mindful eating 6 months after the training program reflected a serious commitment to incorporate what they had learned into their lives. They likely continued using the skills because of the importance of benefits they were experiencing. The decrease in the daily breathing practice from post (70% of participants) to follow-up (39%) may have been a result of a mastery of the skill, feeling less stressed, and thereby having less of a need for a daily breathing practice. The frequency of mindful eating (17 days/month both after the training and at follow-up) was unexpected given the lack of food choices in prison and the quality of food available [38]. A few of the women commented that the mindful eating helped them make better food choices and lose weight, as well as reduce blood sugar in those with diabetes.

This study indicated that it is feasible to hold an MBMTP in the prison environment. Twelve of the trained participants have facilitated at least one 10-week-session mind–body-skills groups with incarcerated peers. One participant has left prison and is now facilitating groups for formerly incarcerated individuals. Leading groups may provide continued benefits since those who are leading the groups are also participating in them and doing the techniques they are teaching, as well as finding satisfaction and meaning in helping others. It is noteworthy that the CMBM faculty observed that the incarcerated women and men who led the small groups in the training did so as sensitively and skillfully as the highly credentialed professionals whom CMBM has trained for more than 30 years.

The large-scale implementation of the program in other prison systems involves certain requirements, including the following:

- Collaborative partnerships—including the commitment of the warden, senior officials, and those responsible for mental health programs in the prison;
- Staff training—having as many wardens, senior officials, and staff as possible attend an MBMTP or workshop prior to offering the MBMTP to the incarcerated. This allows staff to experience the mind–body skills and have a better understanding of the program and its benefits;
- Program sustainability—training staff will ultimately allow them to supervise the incarcerated individuals who will be facilitating mind–body skills groups after the training;
- Resources—the correctional facility must have resources to allocate to the program including appropriate quiet and private space to hold the trainings and mind–body skills groups and the ability to recruit incarcerated individuals for both the MBMTP and the mind–body skills groups they facilitate.

There may also be challenges to developing a mind–body program. In many institutions, time constraints, staffing shortages, and a lack of understanding of the need for staff health promotion can be challenges to developing the program. The benefits of staff wellness, including greater staff retention and work satisfaction, need to be described and evidence for them presented to leadership.

The most obvious limitation of the study, which was a preliminary study to determine the feasibility and effectiveness of the MBMTP in a prison setting, was the lack of a control group. However, given the hardships experienced by incarcerated populations and the resulting ongoing psychological distress, it is unlikely that these improvements would have occurred without the training. A second limitation, inevitable in a prison study during this time period, was the high rate of participants who did not complete the study. One of the inclusion criteria was that participants would not have a sentence that ended or a planned transfer before the study concluded. However, there were considerable delays in starting the trainings, mainly due to COVID-19 outbreaks. Therefore, 8 of the 15 who did not complete the study were either released or transferred. A third limitation is the additional 2-month difference between the initial and advanced trainings for the men compared to the women. Ideally, the two trainings would have been held over the same time frame. However, due to COVD-19 outbreak delays, this was not possible. The fourth limitation was that the frequency of the skills practiced did not include the actual minutes or hours practiced per day, and the results were based on subjective recall. The correlation between the amount of practice and the improvement in outcomes might have been more accurate if it had been possible to collect weekly diaries of practice.

## 5. Conclusions

To our knowledge, this is the first study of a comprehensive training program in prisons that teaches a variety of mind–body and self-care techniques in a supportive small-group setting, and of its capacity to bring about profound change in incarcerated individuals. The results demonstrate that participation in the training resulted in significant improvements in depression, anxiety, and stress, as well as important enhancements in resilience, optimism, belief in their ability to cope, and meaning and purpose in life. However, these findings are preliminary and the results should be interpreted with some caution given the uncontrolled study design. The training provided participants with skills that they could teach their incarcerated peers, and also use to benefit their communities when they leave prison. Many people in the highly stressed prison populations want to learn how to deal with their own stress and trauma and many also want to be helpful to others. This comprehensive training may offer a practical, highly acceptable, sustainable way to support the mental health and the resilience of those in prison, their growth in meaning and purpose, and their prospects for post-prison life. It may well be a model for other correctional facilities which are interested in exploring enhancements in conventional mental health services and expanding opportunities for shaping post-prison lives devoted to service.

## Figures and Tables

**Table 1:** Description of the Mind–Body Medicine Training Program.

Initial Training Sessions ^a^	Topics Taught in the Large-Group Sessions ^b^	Skills Taught in the Small-Group Sessions ^c^
Day 1	Overview of Mind–Body Medicine Introduction to Mind–Body Skills Groups Biological Underpinnings of Mind–Body Therapies	Group 1: Drawings (Part 1) Group 2: Autogenics and Biofeedback
Day 2	Guided Imagery Nutrition and Mindful Eating Mobilizing, Transforming, and Celebrating Emotions	Group 3: Guided Imagery Group 4: Dialogue with a Symptom/Problem
Day 3	Genograms Breathing, Movement, and Medical Considerations of Mind–Body Skills Trauma and Transformation Spirituality and Healing	Group 5: Constructing a Genogram (Part 1) Group 6: Constructing a Genogram (Part 2)
Day 4	Body Awareness Panel Discussion: Taking the Next Step for Practicing the Skills and Question and Answer Session	Group 7: Constructing a Genogram (Part 3) Group 8: Drawings (Part 2)
**Advanced Training Sessions ^a^**	**Description of the Large-Group Sessions ^b^**	**Description of the Small Group Sessions ^c^**
Day 1	Guiding Principles for Mind–Body Skills Groups Coaching Sessions on Leading Mind–Body Skills Groups	Group 1: Soft Belly Breathing (faculty-led)
Day 2	Panel 1: Group Fundamentals Panel 2: Working with Difficult Issues	Group 2: Meditation (participant-led) Group 3: Autogenics and Biofeedback (participant-led)
Day 3	Fishbowl: Demonstration of Working with a Group with Volunteer Participants	Group 3: Meditation and Mindful Eating (participant-led) Group 4: Guided Imagery (participant-led)
Day 4	Next Steps: Groups, Workshops, and Supervision	Group 5: Drawings (participant-led)

^a^ In the Initial Training, participants learned the skills and practiced them in small groups. In the Advanced Training, participants learned how to facilitate a small group and practiced facilitating. ^b^ Each training day began with an opening meditation (breathing or mindful movement). The final days of the Initial and Advanced Trainings ended with a closing ceremony. Topics were taught in a lecture format and there were one to two large-group experiential activities each day that corresponded to one of the lecture topics for the Initial Training Sessions. There were large-group experiential activities on Days 1 and 4 of the Advance Training sessions. ^c^ The basic structure for each of the small groups was as follows: (1) beginning with an opening meditation such as breathing, (2) check-in, (3) explanation of a mind–body technique and practice of the technique, (4) sharing the practice experience with the group, and (5) concluding with a breathing meditation.

**Table 2:** Baseline characteristics of study participants.

Characteristics	Values Are *n* (%) or M (SD)
Gender	
Male	26 (56.5%)
Female	20 (43.5%)
Race	
White	28 (63.6%)
Black	11 (25.0%)
Mixed Race	2 (4.5%)
Other	3 (6.8%)
Ethnicity	
Not Hispanic/Not Latinx	44 (97.8%)
Hispanic/Latinx	1 (2.2%)
Age in Years (*n* = 46)	38.2 (8.9)
Length of Sentence in Years—All Participants (*n* = 46)	24.5 (34.9)
Length of Sentence in Years—Males (*n* = 26)	15.7 (16.4)
Length of Sentence in Years—Females (*n* = 20)	38.8 (47.9)
Time Served in Years—All Participants (*n* = 26)	7.2 (8.6)
Time Served in Years—Males (*n* = 26)	7.5 (7.4)
Time Served in Years—Females (*n* = 20)	6.9 (9.5)
Time Remaining on Sentence in Years—All Participants (*n* = 45)	17.9 (30.6)
Time Remaining on Sentence in Years—Males (*n* = 26)	8.8 (9.6)
Time Remaining on Sentence in Years—Females (*n* = 19)	30.8 (43.2)

Notes: Participants had an age range of 24–57 years. The range of their sentence length was 1.1–215 years. The range of time served at baseline was 3 months to 33 years. The range of time remaining on their sentence length was 10 months to 193 years.

**Table 3:** Results of outcome measures.

Mean Score ^a^ [95% Confidence Interval]					Score Change [95% Confidence Interval]
Outcome Measure	Baseline (T0) (*n* = 45) ^b^	Post Training (T1) (*n* = 31) ^c^	Follow-Up (T2) (*n* = 23)	*p* Value ^d^	Pairwise Comparisons ^e^
CD-RISC (Resilience)	70.2 [66.1, 74.4]	84.6 [81.0, 88.2]	83.6 [78.1, 89.2]	<0.001	T0 vs. T1: 14.3 [9.6, 19.0], *p* < 0.001 T0 vs. T2: 13.4 [6.1, 20.7], *p* < 0.001 T1 vs. T2: −0.9 [−7.0, 5.2], *p* = 0.973
DASS-21: Depression	6.4 [5.0, 7.9]	1.5 [0.6, 2.3]	2.7 [0.8, 4.7]	<0.001	T0 vs. T1: −5.0 [−6.9, −3.1], *p* = <0.001 T0 vs. T2: −3.7 [−6.1, −1.3], *p* = 0.002 T1 vs. T2: 1.3 [−0.9, 3.5], *p* = 0.382
DASS-21: Anxiety	5.3 [4.2, 6.3]	3.0 [2.0, 3.9]	3.5 [2.4, 4.7]	0.006	T0 vs. T1: −2.3 [0.6, 3.9], *p* = 0.004 T0 vs. T2: −1.7 [0.1, 3.5], *p* = 0.048 T1 vs. T2: 0.6 [−0.8, 1.9], *p* = 0.672
DASS-21: Stress	7.6 [6.5, 8.8]	4.1 [2.9, 5.4]	4.7 [3.0, 6.4]	<0.001	T0 vs. T1: −3.5 [−5.5, −1.5], *p* < 0.001 T0 vs. T2: −2.9 [−5.1, −0.7], *p* = 0.006 T1 vs. T2: 0.6 [−1.7, 2.8], *p* = 0.901
Life Orientation Test-R (Optimism)	13.3 [11.7, 14.9]	17.3 [15.7, 19.0]	17.0 [15.2, 18.9]	<0.001	T0 vs. T1: 4.1 [2.0, 6.1], *p* < 0.001 T0 vs. T2: 3.7 [1.3, 6.2], *p* = 0.001 T1 vs. T2: −0.3 [−2.1, 1.5], *p* = 0.968
Coping Self-Efficacy: Total Score	160.5 [145.9, 175.2]	217.0 [204.5, 229.5]	210.3 [191.5, 229.1]	<0.001	T0 vs. T1: 56.5 [36.4, 76.6], *p* < 0.001 T0 vs. T2: 50.0 [25.3, 74.3], *p* < 0.001 T1 vs. T2: −6.7 [−25.7, 12.5], *p* = 0.760
Coping Self-Efficacy: Problem-Focused	6.2 [5.7, 6.8]	8.3 [7.8, 8.9]	8.2 [7.2, 9.0]	<0.001	T0 vs. T1: 2.1 [1.2, 2.9], *p* < 0.001 T0 vs. T2: 1.9 [0.8, 3.0], *p* < 0.001 T1 vs. T2: −0.2 [−1.1, 0.8], *p* = 0.952
Coping Self-Efficacy: Emotion-Focused	5.7 [4.9, 6.5]	8.4 [7.7, 9.2]	7.9 [7.0, 8.7]	<0.001	T0 vs. T1: 3.0 [1.6, 4.4], *p* < 0.001 T0 vs. T2: 2.4 [0.9, 3.8], *p* < 0.001 T1 vs. T2: −0.7 [−1.7, 0.4], *p* = 0.299
Coping Self-Efficacy: Social Support	6.3 [5.5, 7.0]	8.5 [7.8, 9.1]	8.2 [7.3, 9.2]	<0.001	T0 vs. T1: 2.2 [1.2, 3.2], *p* < 0.001 T0 vs. T2: 2.0 [0.8, 3.1], *p* <0.001 T1 vs. T2: −0.2 [−1.1, 0.7], *p* = 0.923
Meaning in Life Questionnaire: Presence of Meaning	23.3 [21.0, 25.7]	29.5 [27.2, 31.7]	28.8 [26.4, 31.2]	<0.001	T0 vs. T1: 6.2 [3.1, 6.2], *p* < 0.001 T0 vs. T2: 5.5 [1.9, 6.0], *p* < 0.001 T1 vs. T2: −0.7 [−2.3, 1.0], *p* = 0.675
Meaning in Life Questionnaire: Search for Meaning	27.1 [25.1, 28.9]	26.3 [23.2, 29.3]	24.8 [20.5, 29.1]	0.555	No pairwise comparisons Change over time was not significant
Life Engagement Test (Purpose in Life)	22.3 [20.9, 23.8]	25.8 [24.3, 27.3]	26.2 [24.9, 27.6]	<0.001	T0 vs. T1: 3.5 [1.5, 6.0], *p* < 0.001 T0 vs. T2: 3.9 [1.5, 6.4], *p* < 0.001 T1 vs. T2: 0.4 [−1.5, 2.3], *p* = 0.921

^a^ Mean scores are Estimated Marginal Means from the repeated measures linear mixed models. ^b^ for Life Engagement, *n* = 46. ^c^ for Resilience, *n* = 30. ^d^ *p* value is for differences across the three timepoints. ^e^ Change scores calculated using the T0, T1, and T2 values on the table may vary slightly compared to those shown in pairwise comparisons due to rounding of the mean scores.

**Table 4:** Change in practice of mind–body skills after mind–body medicine training to follow-up.

Mind–Body Skill	Days per Month (Median)		*z* Score ^a^	*p* Value
	Post Training (T1) (*n* = 23)	Follow-Up (T2) (*n* = 23)		
Soft Belly Breathing	30.4	21.7	--	0.001 ^b^
Meditation	30.4	30.4	--	0.227 ^b^
Mindful Eating	10.9	21.7	0.063	0.950
Dialogue with a Symptom/Problem	4.3	4.3	−0.525	0.600
Guided Imagery	10.9	10.9	−0.974	0.330
Movement (Shaking and Dancing)	10.9	4.3	−2.56	0.010
Biofeedback/Autogenics	4.3	1.0	−0.308	0.758

^a^ The sign test was used for soft belly breathing and meditation so there is no *z* score. ^b^ Exact *p* values from the sign test (used for soft belly breathing and meditation) are based on the binomial distribution because there were less than 25 positive and negative median differences.

**Table 5:** Correlation of mind–body skills practice with improvement in outcomes.

Mind–Body Skills and Outcome Measures	*n*	Timepoint of Frequency of Practice of Mind–Body Skill (T1 = Post Training) (T2 = Follow-Up)	Change in Outcome Measure (T0 = Baseline) (T1 = Post Training) (T2 = Follow-Up)	Correlation Coefficient *r_s_*	*p* Value
Soft Belly Breathing					
CD-RISC (Resilience)	30	T1	T0 to T1	0.410	0.024
DASS-21: Depression	23	T2	T0 to T2	−0.429	0.041
DASS-21 Stress	23	T2	T0 to T2	−0.435	0.038
Life Engagement Test (Purpose in Life)	23	T2	T0 to T2	0.491	0.017
Meditation					
Life Engagement Test (Purpose in Life)	23	T2	T0 to T2	0.503	0.015

## Data Availability

The original contributions presented in this study are included in the Supplementary Materials. Further inquiries can be directed to the corresponding author.

## Supplementary Materials

The following supporting information can be downloaded at: https://www.mdpi.com/article/10.3390/healthcare14060746/s1, Table S1: Practice of mind–body skills; Table S2: Baseline differences of completers vs. non-completers; Dataset: Mind–body medicine training for incarcerated dataset; Supporting Documentation for Dataset: Supporting documentation for the mind–body medicine training for incarcerated dataset; Metadata for Dataset: Metadata for the mind–body medicine training for incarcerated dataset.

## Abbreviations

The following abbreviations are used in this manuscript:

CD-RISC	Connor–Davidson Resilience Scale
DASS-21	Depression Anxiety and Stress Scale-21
MBMTP	Mind–body medicine training program
MBCT	Mindfulness-Based Cognitive Therapy
MBSR	Mindfulness-Based Stress Reduction
LOT-R	Revised Life Orientation Test
MLQ-P	Presence of Meaning in Life Subscale
MLQ-S	Search for Meaning in Life Subscale
CMBM	The Center for Mind–Body Medicine
CSES	Coping Self-Efficacy Scale
IDOC	Indiana Department of Correction
PTSD	Post-traumatic stress disorder
ACT	Acceptance and Commitment Therapy
DBT	Dialectical Behavior Therapy
FCS	Fully conditional specification
LET	Life Engagement Test
MLQ	Meaning in Life Questionnaire
T0	Baseline measurement
T1	Measurement after Advanced Training
T2	Measurement at follow-up
TM	Transcendental Meditation
US	United States
