# Supplementary material for: Mind–Body Medicine Training for Incarcerated Men and Women
Source: Healthcare (Basel). 2026 Mar 16;14(6):746. doi: 10.3390/healthcare14060746 (PMC13027009; doi:10.3390/healthcare14060746)
Supplement: Supplementary file 1 [file healthcare-14-00746-s001.zip › Metadata for Mind body medicine training for incarcerated dataset.pdf]

Metadata for Mind-Body Medicine Training for Incarcerated Dataset

Variable Name	Variable Description	Value Description (where applicable)	Level of measurement
ID	Identification Number		Nominal
Time	Data Collection Timepoint	1= Baseline<br>2 = After mind-body medicine training<br>3 = 6 month follow-up	Ordinal
Sex	Sex	0 =Female<br>1= Male	Nominal
Age	Age		Ratio
AgeCategory	Categories of Age	1 = 20's<br>2= 30's<br>3= 30"s<br>4 = 50's	Ordinal
Ethnicity	Ethnicity	1= Hispanic<br>2 = Non Hispanic	Nominal
Race	Race	1 = White<br>2= Black<br>3 = Other	Nominal
Timeserved Years	Time Served on Prison Sentence in Years		Interval
TimeservedCategory	Time Served on Prison Sentence Categories	1= 5 years or less<br>2 = 6 to 19 years<br>3 = 20 or more years	Ordinal
Resilience	Total Connor Davidson Resilience Scale (CD-RISC) score		Interval
Depression	Depression subscale score of Depression Anxiety and Stress Scale -21(DASS-21)		Interval
Anxiety	Anxiety subscale score of Depression Anxiety and Stress Scale -21 (DASS-21)		Interval
Stress	Stress subscale score of Depression Anxiety and Stress Scale -21(DASS-21)		Interval
CopingSelfEfficacy	Total Coping Self-Efficacy Scale (CSES) score		Interval
ProblemFocused	Problem-Focused Coping subscale score of the CSES		Interval
EmotionFocused	Emotion-Focused Coping subscale score of the CSES		Interval
SocialSupport	Social Support Coping subscale score of the CSES		Interval

PresenceMeaning	Presence of Meaning in Life subscale score of the Meaning in Life Questionnaire (MLQ)		Interval
SearchMeaning	Presence of Meaning in Life subscale score of the Meaning in Life Questionnaire (MLQ)		Interval
OptimismLOT	Total score of the Revised Life-Orientation Test (LOT-R)		Interval
LifeEngagement	Total score of the Life Engagement Test (LET)		Interval
Breathing	Practice of soft belly breathing	0= Never<br>0.5= Less than once per month<br>1 = Once a month<br>2= Twice a month<br>4.3 = Once a week<br>10.85= 2-3 times a week<br>21.7=4-6 times a week<br>30.4=Daily	Ratio
Breathingdifference	Difference in the frequency of practice of soft belly breathing from after the training to follow-up		Ratio
Meditation	Practice of meditation	0= Never<br>0.5= Less than once per month<br>1 = Once a month<br>2= Twice a month<br>4.3 = Once a week<br>10.85= 2-3 times a week<br>21.7=4-6 times a week<br>30.4=Daily	Ratio
Meditationdifference	Difference in the frequency of practice of meditation from after the training to follow-up		Ratio
Imagery	Practice of guided imagery	0= Never<br>0.5= Less than once per month<br>1 = Once a month<br>2= Twice a month<br>4.3 = Once a week<br>10.85= 2-3 times a week<br>21.7=4-6 times a week<br>30.4=Daily	Ratio
Imagerydifference	Difference in the frequency of practice of guided imagery from after the training to follow-up		Ratio
Movement	Practice of movement (shaking and dancing)	0= Never<br>0.5= Less than once per month<br>1 = Once a month	Ratio

		2= Twice a month<br>4.3 = Once a week<br>10.85= 2-3 times a week<br>21.7=4-6 times a week<br>30.4=Daily	
Movementdifference	Difference in the frequency of practice of movement from after the training to follow-up		Ratio
Eating	Practice of mindful eating	0= Never<br>0.5= Less than once per month<br>1 = Once a month<br>2= Twice a month<br>4.3 = Once a week<br>10.85= 2-3 times a week<br>21.7=4-6 times a week<br>30.4=Daily	Ratio
Eating difference	Difference in the frequency of practice of mindful eating from after the training to follow-up		Ratio
Dialogue	Practice of dialogue with a symptom/problem	0= Never<br>0.5= Less than once per month<br>1 = Once a month<br>2= Twice a month<br>4.3 = Once a week<br>10.85= 2-3 times a week<br>21.7=4-6 times a week<br>30.4=Daily	Ratio
Dialoguedifference	Difference in the frequency of practice of dialogue with a symptom or problem from after the training to follow-up		Ratio
Biofeedback	Practice of Biofeedback/Autogenics	0= Never<br>0.5= Less than once per month<br>1 = Once a month<br>2= Twice a month<br>4.3 = Once a week<br>10.85= 2-3 times a week<br>21.7=4-6 times a week<br>30.4=Daily	Ratio
Biofeedbackdifference	Difference in the frequency of practice of biofeedback/Autogenics from after the training to follow-up		Ratio
