# Supplementary material for: Mind–Body Medicine Training for Incarcerated Men and Women
Source: Healthcare (Basel). 2026 Mar 16;14(6):746. doi: 10.3390/healthcare14060746 (PMC13027009; doi:10.3390/healthcare14060746)
Supplement: Supplementary file 1 [file healthcare-14-00746-s001.zip › Supporting Documentation for mind body Medicine training for incarcerated dataset.pdf]

Supporting Documentation for Mind-Body Medicine Training for Incarcerated Dataset

The dataset is in a “long” format with the value of Time as the measurement at each timepoint as defined in the Metadata.

The length of prison sentence and time remaining on the sentence have been omitted from the dataset as they could be considered identifying information for those with very long prison sentences.
