# Supplementary material for: Mind–Body Medicine Training for Incarcerated Men and Women
Source: Healthcare (Basel). 2026 Mar 16;14(6):746. doi: 10.3390/healthcare14060746 (PMC13027009; doi:10.3390/healthcare14060746)
Supplement: Supplementary file 1 [file healthcare-14-00746-s001.zip › Table S1 Practice of Mind-Body Skills.pdf]

Table S1. *Practice of mind-body skills*

**Mind-Body Skill and Time Point**	**Never** *n* **(%)**	**Less than Once per Month** *n* **(%)**	**Once per Month** *n* **(%)**	**Twice per Month** *n* **(%)**	**Once per Week** *n* **(%)**	**2–3 Times per Week** *n* **(%)**	**4–6 Times per Week** *n* **(%)**	**Daily** *n* **(%)**	**Mean (*SD*) Days per Month**
Soft Belly Breathing									
T1	0 (0)	0 (0)	0 (0)	0 (0)	1 (4.3)	2 (8.7)	4 (17.4)	16 (69.6)	26.1 (7.7)
T2	0 (0)	2 (8.7)	0 (0)	1 (4.3)	2 (8.7)	4 (17.4)	5 (21.7)	9 (39.1)	19.0 (11.4)
Meditation									
T1	0 (0)	1 (4.3)	0 (0)	0 (0)	1 (4.3)	3 (13.0)	1 (4.3)	17 (73.9)	25.0 (9.9)
T2	0 (0)	2 (8.7)	1 (4.3)	1 (4.3)	2 (8.7)	1 (4.3)	2 (8.7)	14 (60.9)	21.4 (12.6)
Mindful Eating									
T1	2 (8.7)	0 (0)	0 (0)	0 (0)	3 (13.0)	7 (30.4)	3 (13.0)	8 (34.8)	17.3 (11.3)
T2	0 (0)	2 (8.7)	0 (0)	1 (4.3)	3 (13.0)	4 (17.4)	5 (21.7)	8 (34.8)	17.9 (11.6)
Dialogue with a Symptom/Problem									
T1	2 (8.7)	2 (8.7)	1 (4.3)	1 (4.3)	6 (26.1)	2 (8.7)	6 (26.1)	3 (13.0)	11.7 (11.1)
T2	5 (21.7)	2 (8.7)	2 (8.7)	2 (8.7)	3 (13.0)	0 (0)	4 (17.4)	5 (21.7)	11.2 (12.9)
Guided Imagery									
T1	0 (0)	2 (8.7)	0 (0)	0 (0)	2 (8.7)	9 (39.1)	5 (21.7)	5 (21.7)	16.0 (10.0)
T2	3 (13.0)	0 (0)	1 (4.3)	1 (4.3)	5 (21.7)	3 (13.0)	5 (21.7)	5 (21.7)	13.8 (11.8)
Movement (Shaking and Dancing)									
T1	2 (8.7)	0 (0)	2 (8.7)	0 (0)	7 (30.4)	4 (17.4)	2 (8.7)	6 (26.1)	13.1 (12.0)
T2	3 (13.0)	6 (26.1)	0 (0)	0 (0)	6 (26.1)	5 (21.7)	1 (4.3)	2 (8.7)	7.2 (9.1)
Biofeedback/ Autogenics									
T1	6 (26.1)	4 (17.4)	0 (0)	1 (4.3)	7 (30.4)	3 (13.0)	1 (4.3)	1 (4.3)	5.2 (7.6)
T2	9 (13.1)	1 (4.3)	2 (8.7)	3 (13.0)	2 (8.7)	3 (13.0)	1 (4.3)	2 (8.7)	5.7 (9.4)

Note. *N* = 23; T1 = Post training; T2 = Follow-up
