# Supplementary material for: Mind–Body Medicine Training for Incarcerated Men and Women
Source: Healthcare (Basel). 2026 Mar 16;14(6):746. doi: 10.3390/healthcare14060746 (PMC13027009; doi:10.3390/healthcare14060746)
Supplement: Supplementary file 1 [file healthcare-14-00746-s001.zip › Table S2 Completer vs non-completer.pdf]

Table S2. *Baseline differences of completers versus non-completers*

	Completers (n = 23)[a]		Non-Completers (n = 22)[b]					
**Baseline Outcome Measures and Prison Sentence Demographics**[c]	**Median**	**Mean (SD)**	**Median**	**Mean (SD)**	***t*(43)**	***U***	***z***	***p* value**[c]
CD-RISC (Resilience)	65	68.4 (12.7)	73.5	72.5 (15.2)	1.094	--	--	.280
DASS-21 Depression	8.0	7.2 (4.7)	3.0	5.4 (4.7)	--	304	1.164	.244
DASS-21 Anxiety	5.0	5.6 (3.9)	3.5	4.6 (3.4)	-.855	--	--	.397
DASS-21 Stress	8.0	8.6 (3.7)	5.5	6.3 (3.9)	-2.034		--	.048
Life Orientation Test-R (Optimism)	13.0	13.4 (5.0)	11.0	13.2 (5.9)	--	261	.182	.855
Coping Self-Efficacy: Total Score	155.0	158.7 (49.1)	162.0	165.9 (48.2)	.496	--	--	.623
Meaning in Life Questionnaire: Presence of Meaning	23.0	22.9 (7.3)	25.5	24.2 (8.2)	.564	--	--	.576
Meaning in Life Questionnaire: Search for Meaning	30.0	29.3 (4.7)	25.0	24.7 (6.9)	-2.606	--	--	.013
Life Engagement Test (Purpose in Life)	22.0	22.0 (4.4)	24.0	22.8 (5.2)	--	232	-.716	.474
Length of Prison Sentence	20.0	35.9 (45.5)	10.0	13.0 (12.4)	--	372.5	2.374	.018
Time Served on Prison Sentence	7.0	9.7 (9.1)	3.0	5.0 (7.2)	--	388	2.716	.007
Time Remaining on Prison Sentence	13.5	27.9 (41.2)	7.0	8.4 (7.5)	--	355	2.316	.021

[a] n = 23 for all except Time Remaining on Prison Sentence where n = 22.

[b] Due to a mostly incomplete questionnaire from one participant, n = 22 for all baseline outcome measures except the Life Engagement Test and the prison sentence demographics where n = 23.

[c] For the baseline outcome measures, the median and means are the baseline scores. For the prison sentence demographics, the median and means are years.

[d] Where t scores are given, *p* values are from independent-sample t-tests. Where *U* and *z* scores are given, *p* values are from Mann–Whitney U tests.
